# Somatic Mutations within Myocilin due to Aging May Be a Potential Risk Factor for Glaucoma

**DOI:** 10.3390/genes15020203

**Published:** 2024-02-04

**Authors:** Yevgeniy Sazhnyev, Akaash Venkat, Jie J. Zheng

**Affiliations:** 1 Department of Ophthalmology, Stein Eye Institute, David Geffen School of Medicine, University of California, Los Angeles, CA 90095, USA; sazhnyev@g.ucla.edu (Y.S.); avenkat@ucla.edu (A.V.); 2 Department of Ophthalmology, California Northstate University College of Medicine, 9700 West Taron Dr., Elk Grove, CA 95757, USA; 3 Department of Chemistry and Biochemistry, University of California, Los Angeles, CA 90095, USA; 4 Department of Computer Science, Henry Samueli School of Engineering and Applied Science, University of California, Los Angeles, CA 90095, USA; 5 Molecular Biology Institute, University of California, Los Angeles, CA 90095, USA

**Keywords:** somatic mutation, aging, glaucoma

## Abstract

Glaucoma is a chronic optic neuropathy that leads to irreversible vision loss. Aging and family history are the two most important risk factors of glaucoma. One of the most studied genes involved in the onset of open-angle glaucoma is myocilin (MYOC). About 105 germline mutations within MYOC are known to be associated with glaucoma and result in endoplasmic reticulum (ER) stress, which leads to trabecular meshwork (TM) cell death and subsequent intraocular pressure (IOP) elevation. However, only about 4% of the population carry these mutations. An analysis of MYOC somatic cancer-associated mutations revealed a notable overlap with pathogenic glaucoma variants. Because TM cells have the potential to accumulate somatic mutations at a rapid rate due to ultraviolet (UV) light exposure, we propose that an accumulation of somatic mutations within MYOC is an important contributor to the onset of glaucoma.

## 1. Introduction

Glaucoma is the leading cause of irreversible vision loss and blindness around the world, with it affecting approximately 68.6 million individuals [1,2]. It has been estimated that by the year 2040, 111.8 million people will be affected by this chronic optic neuropathy [3], thus emphasizing the importance of studying the underlying mechanisms associated with its pathophysiology. 

Aging is one of the major risk factors of glaucoma; everyone aged 60 and older is at high risk of glaucoma [1,2]. However, a family history of the disease is also known as a risk factor [1,2]. Advances in the field of genetic and genomic research have led to extensive investigations of the genes contributing to glaucoma and have resulted in the identification of 127 associated genomic regions [4,5,6]. The majority of the identified loci are linked with the onset of open-angle glaucoma (OAG), whereas eight genes are associated with primary angle closure glaucoma [4]. The most frequently occurring subtypes of glaucoma are early-onset juvenile OAG (JOAG) and late-onset primary OAG (POAG), together accounting for 74% of all reported incidents [7]. Mutations in the myocilin gene (*MYOC*) were first identified in families with an autosomal dominant inheritance of JOAG and were linked to the *GLC1A* locus on chromosome 1q24.3-q25.2 [5,8,9,10]. Ever since, extensive efforts have been focused on studying myocilin (MYOC), the most associated protein in OAG development [11,12].

MYOC, a trabecular meshwork-inducible glucocorticoid response protein, is expressed in various tissue and cell types including the trabecular meshwork (TM), ciliary body, retina, myocytes, astrocytes, fibroblasts, endothelial cells, and some epithelial cells [11,13,14]. Despite the broad expression profile of MYOC, mutations in the protein are currently known to cause disease only in the eye. Thermodynamically unstable pathologic germline mutations in MYOC lead to its overexpression in TM cells [15,16,17,18], which initiates a cascade of events that lead to glaucoma. Recent studies have illustrated that increasing the concentration of the transforming growth factor-β2 (TGF-β2) induces *MYOC* expression in TM cells [19], resulting in changes to the extracellular matrix (ECM) structure [11]. Modulating the ECM of TM cells has been shown to obstruct aqueous humor outflow and increase intraocular pressure (IOP), subsequently leading to glaucoma [20].

There are 105 known glaucoma-causing *MYOC* variants (www.myocilin.com), the majority of which localize to the olfactomedin (OLF) domain of MYOC [21]. Glaucoma-associated OLF variants compromise the thermal stability of MYOC [22], leading to protein misfolding and a decrease in melting temperature (T_m_) [23], thus reducing protein folding efficiency [22] and promoting the formation of aggregates within the endoplasmic reticulum (ER) of TM cells [20]. This leads to activation of the unfolded protein response (UPR) pathway and subsequent TM cell death due to elevated ER stress [20]. TM cells play an essential role in modulating the aqueous humor outflow from the anterior chamber of the eye, whereas the death of TM cells has been associated with elevated IOP, retinal ganglion cell death, and irreversible loss of vision through OAG progression [24,25]. Inherited germline mutations in *MYOC* are disease-causing in about 4% of POAG and up to 36% of JOAG cases [26,27], which amount to over 3 million and up to 29 million affected individuals, respectively. A recent study shows a moderate correlation between the stability of inherited germline *MYOC* variants and the age at glaucoma diagnosis [28], which underlines the importance of the thermodynamic stability of mutated *MYOC* in activating the UPR pathway.

Herein, we analyze germline and somatic *MYOC* mutations obtained from glaucoma and cancer genomics databases. *MYOC* genome analysis revealed a remarkable overlap between glaucoma-causing and cancer-associated mutations. Hence, we reason that somatic mutations within *MYOC* may also contribute to glaucoma because an increase in the frequency of ultraviolet (UV) light exposure to the eye can accelerate the accumulation of disease-causing somatic mutations within the *MYOC* of TM cells. This would result in the aging-related pathogenic phenotype of glaucoma. As mutated C→T variants account for ≥60% of UV-induced mutations [29], when combined with the complement G→A variants, *MYOC* may accumulate a large variety of pathogenic mutations due to UV exposure. Indeed, it has been shown that extended periods of time spent outdoors is associated with a higher risk of developing exfoliation syndrome glaucoma [30], underscoring the potential importance of somatic *MYOC* variants in OAG development. Other studies have ascertained a link between age-related neurodegeneration and the accumulation of somatic mutations in neurons [31,32]. Age-associated defects in development and neurogenesis could result from an elevated frequency of somatic mutations due to a higher rate of cell division and an increase in oxidative damage [33]. Thus, we propose that age-related *MYOC* somatic mutations are also major contributors to the onset of glaucoma, and the mechanisms driving the emergence of glaucoma have both hereditary and crucial environmental components. 

## 2. Materials and Methods

### 2.1. Methods for the Mutation Diagram in Figure 1 and Protein Structure Assembly

We obtained 278 known *MYOC* variants that exhibit Mendelian inheritance from the MYOC allele-specific glaucoma phenotype online database (www.myocilin.com, accessed on 10 June 2018); of the 278 *MYOC* germline mutations, 105 manifest a glaucoma phenotype, while 173 were identified as neutral polymorphisms or mutations with unknown pathogenicity. We also found 155 somatic cancer-associated *MYOC* missense and nonsense mutations acquired from the cancer genomics online database cBioPortal (www.cbioportal.org, accessed on 10 June 2018). All of the mutations are listed in the file Glaucoma-Cancer Myocilin Mutation Correlation—Colorcoded.xlsx (Supplementary Information). We utilized the mutation diagram generated through the cBioPortal for Cancer Genomics MutationMapper and modified it using the Hypertext Markup Language (HTML).

**Figure 1:**
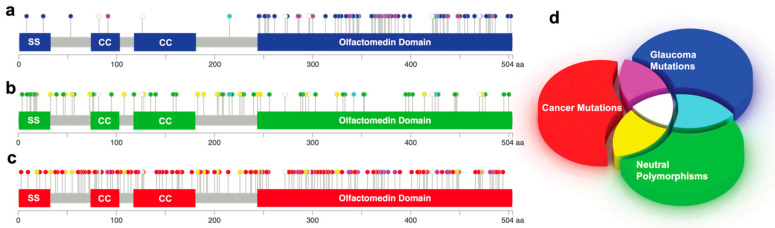
Schematic diagrams of *MYOC* germline and somatic mutations color-coded based on the additive color model. (**a**–**c**) Diagrams depicting MYOC missense and nonsense mutations that are color-coded based on the additive color wheel and represent glaucoma-causing mutations (**a**), neutral polymorphisms or variants of uncertain pathogenicity (**b**), and cancer-associated variants (**c**). SS is defined as the signal sequence and CC is the coiled-coil domain. (**d**) Venn diagram of the additive color model representing mutation phenotypes assigned to a specific color. The overlap of colored cylindrical slices illustrates respective mutation types that are consistent on the mutation diagrams (**a**–**c**).

### 2.2. Methods for Energy-Based Analysis

The MYOC structure (PDB entry 4WXQ) was generated using PyMOL 2.5 computer software for molecular visualization.

Python programming language and the PyRosetta-166 program (www.pyrosetta.org, accessed on 10 June 2018) were used for the Rosetta energy calculations. After obtaining an initial score for the relaxed wild-type protein, the mutate_residue() function within PyRosetta was used to introduce a mutation of interest, and the fast relax on this mutation was also applied. Then, a Cartesian version of Rosetta’s ΔΔG protocol with the β_nov16_cart energy score function was used to calculate the ΔΔG values of the mutations from the wild type.

## 3. Results

The *MYOC* gene is mapped to chromosome 1q24.3-q25.2 and comprises three exons that encode a 58 kD, 504 amino acid polypeptide with a leucine zipper motif within a coiled-coil domain at the N-terminus and an OLF domain at the C-terminal fragment [34] (Figure 1a–c). The N-terminal segment includes a signal sequence at amino acid position 1–32, which is cleaved off at the ER [11,35] during protein synthesis. Recent evidence suggests that the homo-oligomerizing coiled-coil domains at amino acids 74–102 and 118–180 assemble into a Y-shaped tripartite parallel dimer of dimers structure [36]. Glaucoma-causing variants within the tetramer modulate the structure without altering stability [36]. MYOC undergoes proteolytic cleavage between residues R226 and I227, which produces a 35 kD secreted C-terminal OLF domain and a 20 kD N-terminal tetramer fragment with coiled-coil domains [37]. The OLF domain comprises amino acids 244–503 and contains two cysteine residues that participate in disulfide bond formation, C245 and C433 [11,38].

We obtained 278 known *MYOC* variants that exhibit Mendelian inheritance from the MYOC allele-specific glaucoma phenotype database [21]; of the 278 *MYOC* germline mutations, 105 manifest a glaucoma phenotype, while 173 were identified as neutral polymorphisms or mutations with unknown pathogenicity [21]. Using small cycles, all of the mutations are summarized in Figure 1. The colors of those cycles are defined in the Venn diagram (Figure 1d). The overlaps between glaucoma-causing and neutral polymorphisms (cyan cycles) are the residue positions where the mutated amino acids are different.

We also found 155 somatic cancer-associated *MYOC* missense and nonsense mutations acquired from the cancer genomics online database, cBioPortal (www.cbioportal.org) [39,40]; the most frequent cancer types associated with these mutations are cutaneous melanoma, glioblastoma multiforme, and uterine endometrioid carcinoma. Notably, 38 glaucoma-causing germline mutations (36%) [21] and 38 melanoma somatic variants (83% of the total melanoma mutants) [39,40] arise from C→T or G→A nucleotide transitions. There are 11 MYOC somatic mutations that precisely overlap with glaucoma-inducing germline mutations (R91STOP, G246R, L255P, T285M, R296C, A363T, G367R, T377M, D384N, A427T, and R470C). All white circles within the mutation diagrams demonstrate varying amino acid changes at the same position with exact overlaps between either cancer and glaucoma variants or cancer mutations and neutral polymorphisms.

**Figure 2:**
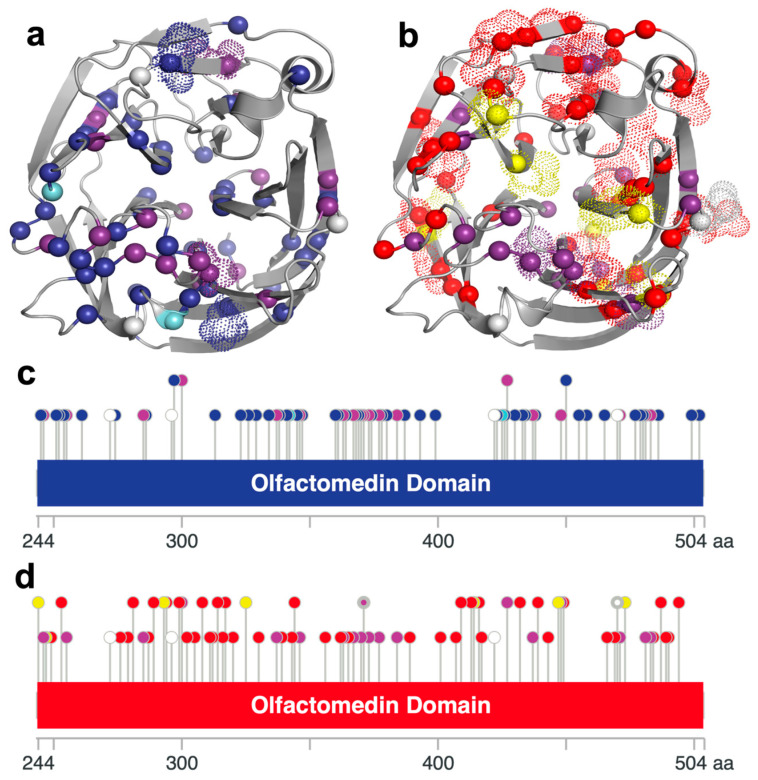
Graphical representation of MYOC cancer-associated somatic and glaucoma-causing germline variants color-coded according to the Venn diagram additive color model (Figure 1d). (**a**,**b**) Cartoon molecular structures of the MYOC OLF domain portraying glaucoma—(**a**) and cancer-associated (**b**) mutations as *α*-carbon spheres. Stabilizing mutations are illustrated as dotted Van der Waals structures. (**c**,**d**) MYOC OLF domain diagrams depicting glaucoma-causing (**c**) and cancer-linked (**d**) variants with positive ΔΔG and negative ΔΔG values plotted on the bottom (unstable variants) and top row (stable variants), respectively. The stabilizing R470H cancer-linked and neutral variant, illustrated as a white circle with a thicker lining (**c**), overlaps with a glaucoma-causing and cancer-inducing R470C mutation, which did not exhibit favorable thermodynamic properties.

Somatic cancer-associated variants and germline neutral polymorphisms are spread evenly across the entire protein with frequent overlap; glaucoma-causing germline mutations largely localize to the OLF domain of MYOC (Figure 1a,b). All except one glaucoma and cancer overlapping variant localized to the OLF domain. To investigate the thermodynamic parameters of MYOC and determine the effect of each mutation on protein stability, we utilized the Rosetta energy function and calculated the change in free energy (ΔΔG) of individual cancer and glaucoma mutations [41] (online methods) with refinement (β_nov16_cart), which provided a very efficient sampling of the global minimum [41]. As expected, stabilizing mutations were predominantly located on the exterior of the MYOC OLF domain. These variants are schematically illustrated as dotted sidechain structures for glaucoma-causing (Figure 2a) and cancer-associated (Figure 2b) mutations. Amino acid changes are color-coded based on the Venn diagram additive color model (Figure 1a). The stable and unstable variants of glaucoma-causing and cancer-linked mutations within the MYOC OLF domain are also shown in Figure 2c and Figure 2d, respectively; in the figures, variants with positive and negative ΔΔG values are plotted on the bottom and top row, respectively. The ratio of cancer to glaucoma-stabilizing variants was significant; 31 cancer-associated and four glaucoma-inducing mutations exhibited negative ΔΔG values. Five of these mutations overlapped with the germline neutral polymorphisms (T293K, T325M, E414K, A447V, and R470H), and one variant (A427T) overlapped with a germline glaucoma-causing mutation observed in older individuals (61 ± 21.1 years of age) [21]. A427T was previously reported to possess a relatively high T_m_ (48.3 ± 0.3 °C) compared to other glaucoma-causing mutations [23]. Additionally, the other three stabilizing glaucoma-inducing mutations, Q297H, E300K, and N450D, also had a late age onset between 60–75 years of age [21,42].

G367R, a germline glaucoma-causing variant with an early age of onset (13–23 years of age) and a high maximum IOP (40 mmHg) [43] was observed in the tumors of patients with esophageal adenocarcinoma. Interestingly, this variant was not stabilizing according to Rosetta calculations with a ΔΔG value of +4.23 kcal/mol. Several glaucoma pathogenic mutations manifested similar characteristics (i.e., overlap with somatic variants, early age of onset, and destabilizing properties) including G246R, L255P, T285M, R296C, A363T, P370L, T377M, D384N, and R470C, thus recapitulating the likelihood that these pathogenic mutations accumulate within the MYOC of TM cells and largely contribute to the onset of glaucoma.

## 4. Discussion

In this study, we present a collection of known somatic and germline *MYOC* variants and demonstrate that a significant level of overlap exists between the two types of mutations. We propose that an accumulation of pathogenic *MYOC* somatic mutations within the TM of the eye by means of UV radiation throughout aging can lead to protein aggregation, induced ER stress, TM cell death, and subsequent IOP elevation, which are attributes of the glaucoma phenotype induced by the known germline *MYOC* glaucoma-causing variants. In particular, UV-induced somatic C→T or G→A transition mutations within the *MYOC* coding region that lead to pathogenic MYOC missense or nonsense glaucoma variants are likely to be overexpressed in glaucomatous TM cells. Therefore, age-related glaucoma onset may be influenced by somatic mutation buildup within key genes associated with its pathophysiology. 

MYOC mutations can either be detrimental or stabilizing depending on the thermodynamic properties of the mutated residues’ biochemical environment. Deleterious mutations, such as MYOC glaucoma-causing variants, reduce the folding efficiency of a protein and can induce protein aggregation, among other harmful responses. Genome editing using clustered regularly interspaced short palindromic repeats (CRISPR)-Cas9 technology has been shown to accomplish the alleviation of the glaucomatous phenotype by knocking down the expression of mutant MYOC, reducing ER stress, and ultimately decreasing IOP [44]. With the advent of genetic modification, it may soon be feasible to reverse the harmful mutations within the genome and prevent the onset of age-related neurodegenerative diseases. On the other hand, stabilizing mutations can enhance mutated protein’s stability, resulting in a product that can sustain its desired native structure to a higher degree. Indeed, many cancer-associated variants that lead to more stable protein products likely have gain-of-function properties, thereby leading to cancer progression. For example, MYOC mutations that were determined to be stabilizing have been found in prostate neuroendocrine, cervical squamous cell, uterine endometrioid, bladder urothelial, and lung squamous cell carcinomas; colon, endocervical, colorectal, prostate, and lung adenocarcinomas; and acral and desmoplastic melanomas, uterine carcinosarcoma, anaplastic astrocytoma, and diffuse glioma [39,40]. The most stabilizing E253Q variant is associated with endocervical adenocarcinoma, with the next stabilizing A447V mutation linked to colon adenocarcinoma [39,40]. Four of the glaucoma-causing mutations are stabilizing MYOC variants (Figure 2c), and two of them overlap with cancer somatic mutations (Figure 2d). Those mutations may have an alternative pathway for glaucoma progression and are likely not involved in the activation of the UPR pathway. Further studies on those stabilizing glaucoma-causing variants may explore the possibility of uncovering a secondary pathological mechanism of multifaceted glaucoma progression.

## 5. Conclusions

In summary, our analysis suggests that the accumulation of pathogenic somatic variants within individual cells may also contribute to other age-related diseases, as observed in the development of cancers [45]. Additionally, glaucoma-causing MYOC stabilizing mutations may contribute to its pathogenesis via an alternative pathway not involved in ER stress induction. Furthermore, stabilizing MYOC cancer-associated variants could be cancer risk factors for those who carry the mutations.

## Data Availability

All of the data are included in Supplementary Materials.

## Supplementary Materials

The following supporting information can be downloaded at: https://www.mdpi.com/article/10.3390/genes15020203/s1. Glaucoma-Cancer Myocilin Mutation Correlation—Colorcoded.xlsx.
